# Rapid diagnostic testing for onchocerciasis in Maridi (South Sudan) before and after improving elimination strategies: a repeated cross-sectional survey

**DOI:** 10.12688/openreseurope.16093.2

**Published:** 2024-03-22

**Authors:** Amber Hadermann, Stephen Raimon Jada, Luís-Jorge Amaral, Robert Colebunders, Yak Yak Bol, Joseph N Siewe Fodjo

**Affiliations:** 1 Global Health Institute, University of Antwerp, Antwerp, Flanders, 2610, Belgium; 2 Amref Health Africa South Sudan, Juba, South Sudan; 3 Neglected Tropical Diseases Programme, Ministry of Health, Juba, South Sudan

**Keywords:** Onchocerciasis, Onchocerca volvulus, antibodies, transmission, testing, seroprevalence, children, ivermectin, vector control

## Abstract

**Background:**

Maridi County is an onchocerciasis-endemic area in South Sudan. Annual community-directed treatment with ivermectin (CDTi) was instituted in Maridi since the early 2000s, but with low coverage. In 2021, the CDTi programme was strengthened to a six-monthly programme. Additionally, the community-based vector control strategy “Slash and Clear” has been implemented since 2019 at the Maridi Dam, the only blackfly breeding site in the area. This study assessed the effect of these reinforced onchocerciasis elimination interventions on the *Onchocerca volvulus* seroprevalence among young children, an indicator of ongoing transmission.

**Methods:**

Baseline and follow-up serosurveys were conducted in Maridi in 2019 (prior to strengthening onchocerciasis elimination efforts) and 2023, respectively. During both surveys, children aged three to nine years were recruited from five study sites situated at different distances from the Maridi Dam. Ov16 antibodies were detected via rapid diagnostic tests (RDTs) using whole blood obtained by finger-pricking the participants. Baseline and follow-up Ov16 prevalence rates were calculated and compared.

**Results:**

In 2019, the Ov16 seroprevalence among children aged three to nine years was 24.5% compared to 30.6% in 2023 (p=0.22). Both surveys found a particularly high Ov16 seroprevalence in the study site closest to the Maridi Dam (35.0% in 2019 and 44.0% in 2023, p=0.52). The Ov16 seroprevalence had a non-significant decreasing trend in the three-year-old children, from 12.5% (3/24) in 2019 to 8.8% (3/34) in 2023 (p=0.65).

**Conclusion:**

The persistent Ov16 RDT seropositivity among three-year-old children in 2023 indicates ongoing *O. volvulus* transmission. Therefore, further strengthening of the onchocerciasis elimination programme is required. The study highlights the utility of RDTs in monitoring onchocerciasis transmission in highly endemic settings.

## Introduction

Onchocerciasis is a neglected tropical disease primarily found in Africa, caused by the filarial nematode *Onchocerca volvulus* and transmitted through bites of infectious blackflies ( *Simulium* spp.) ^1^. Onchocerciasis is known to cause dermatitis and blindness (“river blindness”) and is associated with a clinical spectrum of seizures and disorders grouped under the term onchocerciasis-associated epilepsy (OAE) ^2^. In areas where there is high ongoing *O. volvulus* transmission, children are at risk of developing OAE, with seizure onset typically occurring between the ages of three to 18 years ^2^. Elimination of onchocerciasis depends mainly on community-directed treatment with ivermectin (CDTi) in at-risk communities, which can be supplemented with activities to eradicate the blackflies ^3^.

Onchocerciasis was first reported in Sudan in 1947 by Kirk *et al.* Ivermectin distribution started in South Sudan in 1996 through the Southern Sudan Onchocerciasis Control Programme, but annual CDTi was only formally initiated in 2005 ^4, 5^. Moreover, there have been several years of low coverage treatment due to insecurity, and community drug distributors' high attrition rate, which, in combination with high onchocerciasis transmission potential, has allowed the disease to remain endemic most of the country ^4– 6^. Although CDTi was interrupted in 2020 because of the COVID-19 pandemic, it has been re-introduced biannually (six-monthly) since 2021.

Currently, the World Health Organisation (WHO) recommends using serodiagnostic tools based on the detection of antibodies specific to a 16KDa species-specific antigen of *O. volvulus* (Ov16), utilising either a rapid diagnostic test or an Enzyme-Linked Immunosorbent Assay (ELISA) platform ^7^. The antigen Ov16 serves as an early marker of onchocerciasis infection, so seroconversion to Ov16 can be used to detect pre-patent infection, which is not possible with skin snips ^8^. Since its discovery in 1991, the use of Ov16 has emerged as the gold standard for onchocerciasis serology, with sensitivities in the 80–89% range and specificity values in the 97–99% range depending on the assay used and the sample collection assessed ^9, 10^.

**Figure 1.**
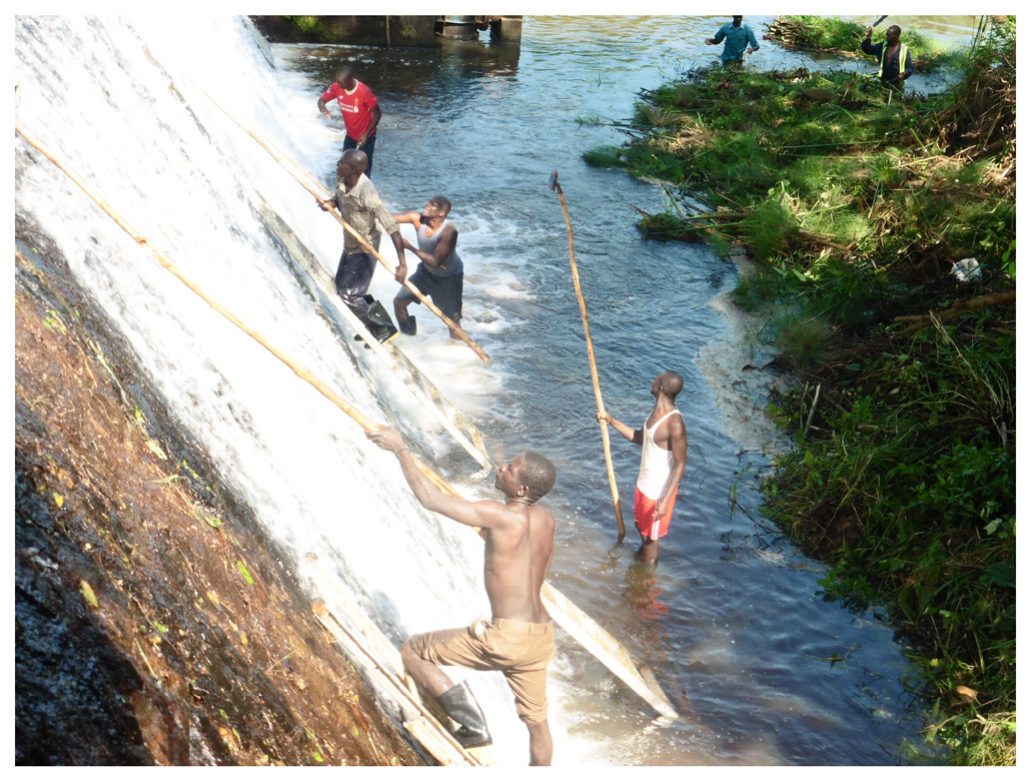
Slash-and-Clear at the Maridi Dam, South Sudan.

In 2018, a house-to-house survey in Maridi County, an onchocerciasis-endemic area in Western Equatoria State, South Sudan, showed a very high epilepsy prevalence of 43.8 per 1000 individuals ^11^. Notably, in Kazana 2, the site closest to the major blackfly breeding site in the area (Maridi Dam), an epilepsy prevalence of 119.0 per 1000 individuals was noted ^11^. In December 2019, onchocerciasis transmission was assessed in Maridi in three sites at different distances from the Maridi Dam via an Ov16 serosurvey in children three to nine years of age. The overall seroprevalence of the children was 24.5% ^12^. As these findings indicated high onchocerciasis transmission, a community-based vector control strategy, “Slash and Clear”, has been implemented at least once a year at the Maridi Dam since December 2019 ( Figure 1) ^13^. This strategy consists of clearing the trailing vegetation to eliminate the blackfly breeding site and reduce biting density in the nearby communities.

In February 2023, a follow-up *O. volvulus* serosurvey was conducted among children aged three to nine years in Maridi to evaluate possible changes in transmission patterns. This paper presented the effect of strengthening the onchocerciasis elimination interventions as demonstrated by seroprevalence in children aged three to nine years using the Ov16 SD BIOLINE rapid diagnostic test (RDT).

## Methods

### Ethical standards

Ethical approval for this study was obtained from the University of Antwerp’s ethics committee (Ref: BUN B300201940004) and from the ethics committee of the Ministry of Health of South Sudan (MOH/RERB 56/2022). All participants were recruited only after informed parental consent was granted (and assent for children aged seven years and above). The research procedures were in compliance with the declaration Helsinki, and all personal data were treated confidentially.

### Study setting

**Figure 2.**
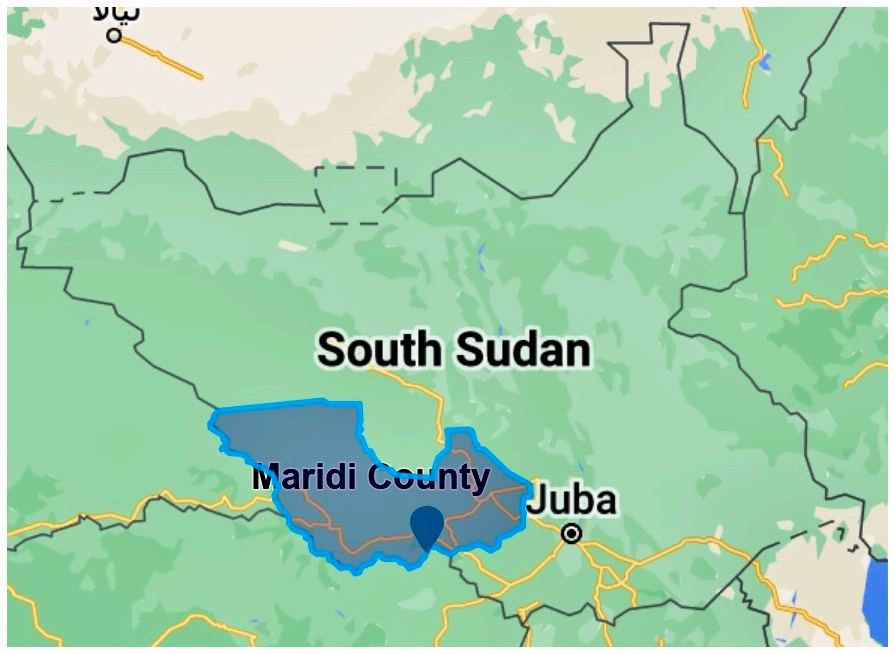
Location of Maridi County in Western-Equatorian State, South Sudan (produced in Scribble Maps).

South Sudan is known to have multiple endemic hotspots of onchocerciasis, including Maridi County in the Western Equatoria State ( Figure 2).

**Figure 3.**
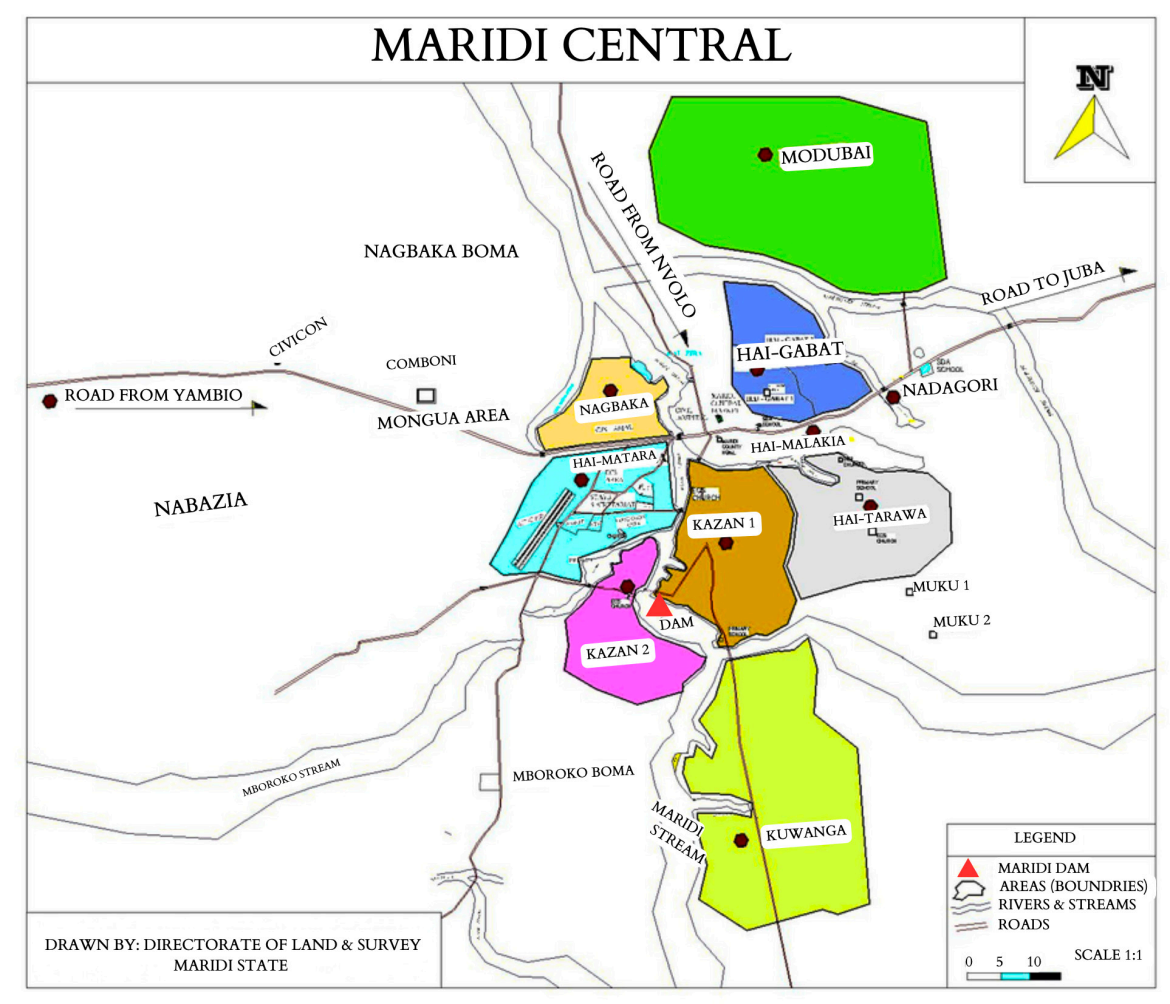
The Maridi central area with the location of high- (Kazana 1 and 2 and Hai-Matara sites) and low-onchocerciasis transmission zones (Hai-Gabat and Hai-Tarawa sites) and the Maridi Dam (adapted from Colebunders *et al*. ^11^).

Maridi County is home to over 115,000 individuals ^14^ and is crossed by the Maridi River, upon which a dam was built in the 1950s. The dam spillway was identified as the sole blackfly breeding site in Maridi ^12^, driving onchocerciasis transmission in the neighbouring villages. This study was conducted between 2019 (baseline) and 2023 (follow-up) in five villages, of which Kazana 1, Kazana 2, and Hai-Matara are situated in the proximity of the dam (high-transmission zone; HTZ) and Hai-Tarawa and Hai-Gabat are located further away from the dam (low-transmission zone; LTZ) ( Figure 3).

### Study procedures

A cross-sectional study was conducted before and after strengthening the control interventions against onchocerciasis. The procedures of the baseline survey (2019) have been described in detail previously ^12^. Several weeks before the follow-up survey in 2023, the research team contacted the community leaders to inform them about the study and obtain their consent and collaboration. Five local healthcare workers were trained to perform the Ov16 SD BIOLINE RDT and administer a questionnaire to the parents of the children. One day before participant recruitment, community mobilisers notified villagers about the study and of the site(s) chosen in each village (schools, churches, etc.) to test the eligible children (three to nine years old). On the day of the study in each study site, the mobilisers used megaphones, and healthcare workers went from house to house to gather more participants. All children for whom informed consent was provided by a parent/guardian were enrolled in the study (assent was obtained from children aged seven to nine years).

A short questionnaire was administered to the parent/guardian and the child to collect relevant information about the child’s history (age, sex, village of residence, previous ivermectin intake, itching/skin lesions, epilepsy). Thereafter, all the participating children were finger-pricked with a single-use, retractable lancet of 2.00 millimetres (Ergo lance). Whole blood from each finger prick was used for Ov16 rapid diagnostic testing (RDT) (SD Bioline, Inc, Gyeonggi-do, South Korea). The Ov16 RDT test was read after 30 minutes and performed in the field following the manufacturers’ instructions.

### Data analysis

Study data was checked daily and uploaded to the REDCap secure online platform. The final datasets were exported from REDCap, cleaned in Excel spreadsheets, then transferred to the R software version 4.2.2 for analysis. Continuous variables were summarised as median with interquartile range (IQR), while categorical variables were expressed as percentages. The findings of the baseline (2019) ^12^ and follow-up (2023) surveys were compared using the Chi-squared test (or Fisher’s exact test when appropriate), and 95% confidence intervals (CIs) were produced using the Wilson score with continuity correction.

## Results

### Ov16 seroprevalence in Maridi 2023

During the 2023 *O. volvulus* seroprevalence study in Maridi, 248 children aged three to nine years were recruited. The median age was six years (IQR: 4–8), and 114 were males (46.0%). Ninety-three of the participating children (37.5%) had some form of dermatitis, as evidenced by itching and/or visible skin lesions. Furthermore, four children (1.6%) were identified as having epilepsy.

**Figure 4.**
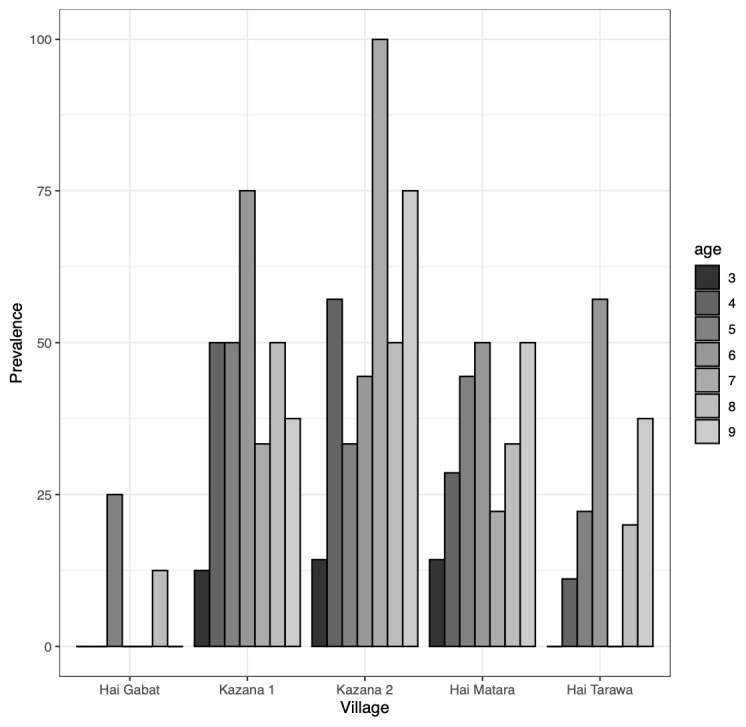
Ov16 rapid diagnostic test seroprevalence in 2023 per study site and age (years old).

The overall Ov16 RDT seroprevalence was 30.7% (76/248). The seroprevalence was significantly different across villages (p<0.001), with Kazana 1 and Kazana 2 being the most affected and Hai-Gabat and Hai-Tarawa less affected ( Figure 4).

The Ov16 RDT seroprevalence was similar for males and females (p=0.98) and did not vary significantly across the younger (30.7% seropositivity among children aged <7 years) and older (30.5% seropositivity among aged ≥7 years) age-groups (p=0.78) ( Table 1). However, the 3-year-olds, had lower Ov16 seropositivity: 8.8% (3/34) versus 34.1% (73/214) in the older children; p<0.001.

**Table 1.** Ov16 rapid diagnostic test seroprevalence in 2023 by age group and sex across included villages.

Ov16 RDT seroprevalence % (Ratio, 95%CI)						Comparisons	
Village	Hai-Gabat	Kazana 1	Kazana 2	Hai-Matara	Hai-Tarawa	Overall	*p*-value *
**Age-groups**							
**3–6 years**	3.4 (1/29, 0.2-19.6)	46.7 (14/30, 28.8-65.4)	43.8 (14/32, 26.0-60.6)	35.5 (11/31, 19.8-54.6)	7/30 (23.3, 10.6-42.7)	30.7 (47/153, 23.7-38.8)	0.98
**7–9 years**	4.8 (1/21, 0.2-25.9)	8/20 (40.0, 20.0-63.6)	66.7 (8/12, 38.9-89.6)	33.3 (7/21, 15.5-57.0)	4/20 (20.0, 0.7-44.3)	30.5 (29/95, 21.7-40.9)	
**Sex**							
**Male**	3.6 (1/28, 0.2-20.2)	8/16 (50.0, 28.0-72.0)	55.0 (11/20, 32.0-76.2)	38.9 (7/18, 18.3-64.0)	6/31 (19.4, 8.1-38.1)	29.2 (33/113, 21.2-38.6)	0.78
**Female**	4.5 (1/22, 0.2-24.9)	14/34 (42.2, 25.2-59.2)	46.6 (12/26, 27.1-66.3)	32.4 (11/34, 18.0-50.6)	5/19 (26.3, 10.1-51.4)	31.9 (43/135, 24.3-40.5)	
**Overall**	30.6 (76/248, 25.1-36.9)						

* All p-values were obtained with a Chi-square test/Fisher’s exact test. CI – confidence interval.

Children with dermatitis had a higher Ov16 RDT seroprevalence (43.5%, 40/92) compared to their peers without dermatitis (22.6%, 35/155); p< 0.001. Of the four children with epilepsy, none tested positive for Ov16 antibodies.

### Ov16 seroprevalence in Maridi over time

**Figure 5.**
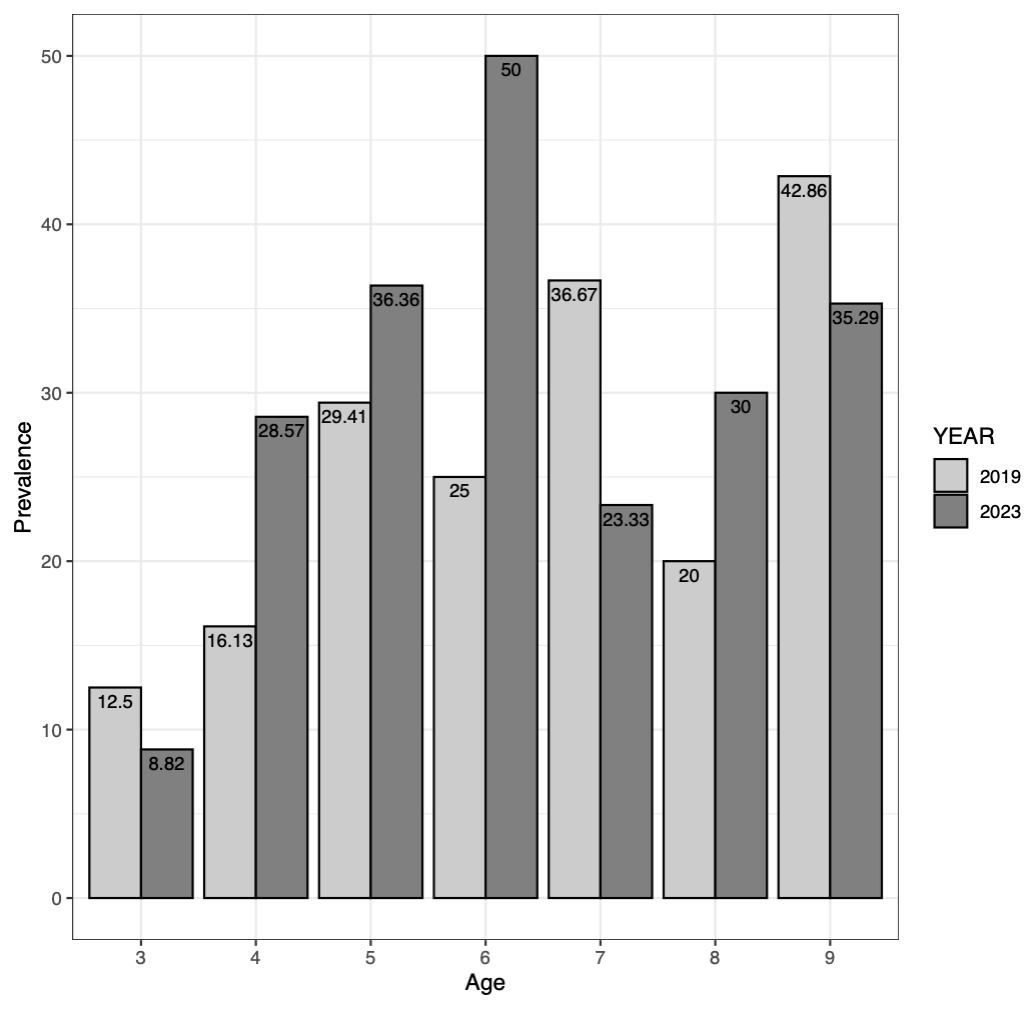
Ov16 rapid diagnostic test seroprevalence (%) by age and year surveyed (2019 and 2023).

Comparing baseline and follow-up Ov16 RDT results, the overall Ov16 seroprevalence increased non-significantly from 24.3% in 2019 to 30.6% in 2023 (p=0.22) ( Figure 5). The seroprevalence among three-year-olds, born after implementation of more robust onchocerciasis elimination measures like vector control and bi-annual CDTi, was measured at 8% in 2023 and was thus lower than the level of 12.5% measured among three-year-olds in 2019. However, the difference was not significant (p=0.68). Conversely, the Ov16 seropositivity rose non-significantly from 26.7% in 2019 to 34.1% in 2023 among children aged four years and above (p=0.16). Transmission zone-specific Ov16 seroprevalence did not significantly change across surveys (HTZ: p= 0.70; LTZ: p= 0.18; Table 2).

**Table 2.** Ov16 rapid diagnostic test seroprevalence among three- to nine-year-olds per survey (2019/2023) and village.

	2019 seroprevalence %(Ratio, 95%CI)	2023 seroprevalence %(Ratio, 95%CI)	*p*-value *
**Village**			
**Hai-Gabat**	0.0 (0/24, 0.0-17.2)	4.2 (2/48, 0.7-14.9)	N.A.
**Kazana 1**	35.0 (14/40, 21.1-51.7)	44.0 (22/50, 30.3-58.7)	0.52
**Kazana 2**	50.0 (12/24, 31.4-68.6)	50.0 (23/46, 36.1-63.9)	1.00
**Hai-Matara**	14.8 (4/27, 4.9-34.6)	34.6 (18/52, 22.3-49.2)	N.A.
**Hai-Tarawa**	17.2 (5/29, 6.5-36.5)	22.0 (11/50, 12.0-36.3)	0.83
**Transmission zones**			
**Low-transmission zone**	9.4 (5/53, 3.5-21.4)	13.0 (13/100, 7.4-21.6)	0.70
**High-transmission zone**	33.0 (30/91, 23.7-43.7)	42.6 (63/148, 34.6-51.0)	0.18
**Overall**	24.3 (35/144, 17.7-32.3)	30.7 (76/248, 25.1-36.9)	0.22

* All p-values were obtained with a Chi-square test/Fisher’s exact test. N.A.: not applicable due to a too-rare event; CI – confidence interval.

## Discussion

In 2023, an Ov16 seroprevalence of 30.7% was found among children aged three to nine years in Maridi, representing a non-significant increase from the 24.3% seroprevalence documented in 2019 ^12^ (p=0.98). As the elimination measures were only introduced three years ago, children between the ages of four and nine years in the 2023 cohort had already been highly exposed to onchocerciasis before strengthening the control interventions. An increase in the seroprevalence in the age group of three to nine years old children was to be expected, particularly because of the very high pre-intervention endemicity ^15^. Indeed, some of the children might harbour prepatent infection before the start of the intervention, which may break out in the course of the intervention. A force-of-infection model for onchocerciasis has shown that upon interrupting *O. volvulus* transmission by vector control, the microfilarial prevalence of the zero to four and five to nine year old age groups continues to rise during the first five years, after which it starts falling ^16^.

The fact that the Ov16 seroprevalence among the three-year-olds did not decrease significantly may also be related to the small sample size, short follow-up duration since the strengthening of the control interventions and the lack of CDTi in 2021. Indeed, mathematical modelling has suggested that a one-year CDTi interruption may significantly impact onchocerciasis elimination prospects in settings such as Maridi, with short CDTi histories and high onchocerciasis endemicity ^17, 18^. The one-year CDTi interruption in 2020 and the consistently low CDTi coverage rates achieved in Maridi (40.8% in 2017 ^11^ and 56.6% in 2021 ^19^) explain why some three-year-old children became infected with *O. volvulus*. Achieving a CDTi coverage of at least 80% of the total population is strongly recommended for the successful elimination of onchocerciasis transmission, particularly in areas with a high prevalence of the disease ^20^.

The high Ov16 seroprevalence among young children in Maridi reveals that the onchocerciasis elimination measures must be further strengthened to protect children from developing onchocerciasis-associated morbidities. Over one-third (37.5%) of the children had some form of dermatitis, as evidenced by itching and/or visible skin lesions. A significantly higher proportion of children with dermatitis tested Ov16 RDT positive (43.5%) compared to their peers without dermatitis (22.6%, p<0.001), suggesting the dermatitis was likely caused by an active *O. volvulus* infection in a large proportion of children. In resource-constrained settings such as many parts of sub-Saharan Africa, when children present with dermatitis and itching, scabies should be considered as a possible diagnosis. However, the absence of characteristic scabies lesions was noted in the children included in the study. Additionally, since scabies is treatable with ivermectin, its prevalence typically decreases in regions where mass ivermectin distribution is implemented ^21^.

This study demonstrates the feasibility of using rapid diagnostic tests for evaluating onchocerciasis transmission in highly endemic settings. Until recently, field Ov16 RDTs were overlooked in monitoring onchocerciasis transmission in endemic foci since the gold standard technique recommended by the World Health Organization (WHO) was Ov16 enzyme-linked immunosorbent assay (ELISA) ^22^. However, in practice, there are logistical challenges to performing Ov16 ELISA (need for a cold chain, specialised equipment, and capacity), particularly in remote sites like Maridi. Meanwhile, studies in Cameroon ^23^ and the Democratic Republic of Congo ^24^ have found that Ov16 RDT done in the field was helpful in investigating onchocerciasis transmission patterns. Therefore, it is worth considering the widespread use of RDTs as a point-of-care approach for monitoring onchocerciasis in resource-limited sites.

Our study had some limitations. A sufficiently large sample size of 3-year-old children needs to be Ov16 RDT tested, and a longer follow-up is needed to investigate effect of strengthening an onchocerciasis elimination programme. Moreover, in the absence of skin snips for microfilariae quantification and blackfly infection rates, it is difficult to obtain precise estimates of the intensity of *O. volvulus* transmission in Maridi. Further research may be required to better understand the Ov16 antibodies dynamics during and after *O. volvulus* infection. Strengthening the onchocerciasis elimination program certainly decreased the *O. volvulus* transmission in Maridi. This was suggested by the results of a recent longitudinal population-based study that showed that the incidence of onchocerciasis-associated epilepsy, including nodding syndrome, considerably decreased in Maridi between 2018 and 2022 ^25^.

In conclusion, using Ov16 RDT, we were were able to demonstrate that Maridi remains a hotspot for *O. volvulus* transmission even after the implementation of elimination measures. These measures should be continued and strengthened even further, including increasing the CDTi coverage to protect children from developing OAE and to move towards the elimination goals of WHO in 2030.

## Data Availability

EUDAT. Rapid diagnostic testing for onchocerciasis in Maridi (South Sudan) before and after improving elimination strategies. DOI: 10.23728/b2share.e98101581be64e6188836d73a19d0243 ^26^. This project contains the following data: - Baseline and follow-up serosurveys were conducted in Maridi in 2019 (prior to strengthening onchocerciasis elimination efforts) and 2023, respectively. During both surveys, children aged three to nine years were recruited from five study sites situated at different distances from the Maridi Dam. Ov16 antibodies were detected via rapid diagnostic tests (RDTs) using whole blood obtained by finger-pricking the participants. Baseline and follow-up Ov16 prevalence rates were calculated and compared. Data are available under the terms of the Creative Commons Zero "No rights reserved" data waiver (CC0 1.0 Public domain dedication).
