# Supplementary material for: Rapid diagnostic testing for onchocerciasis in Maridi (South Sudan) before and after improving elimination strategies: a repeated cross-sectional survey
Source: Open Res Eur. 2024 Mar 22;3:206. Originally published 2023 Nov 21. [Version 2] doi: 10.12688/openreseurope.16093.2 (PMC11016167; doi:10.12688/openreseurope.16093.2)
Supplement: Peer review history

# Reviewer report: Reviewer response for version 2

**DOI:** 10.21956/openreseurope.18760.r39104

**Published:** 2024-04-22

**Authors:** Yankum Dadzie

**Affiliations:** 1 Emory University, Atlanta, Georgia, USA

The paper reads well and the discussion has been improved significantly. As it has been implied, the impact of the additional intervention measures which need to be strengthened will be apparent in a few more years.

 However, the discussion on the use and attributes of Ov 16 RDT as a diagnostic tool includes a statement that: ‘until recently, the RDT tool has been overlooked in the monitoring of onchocerciasis transmission in endemic foci since WHO recommended the use of ELISA as in reference 22 of this paper'.  This statement, however, is misleading in the sense that the WHO Guidelines which the reference 22 refers, discusses only the modalities required to demonstrate that interruption of onchocerciasis transmission has occurred and therefore mass drug administration (MDA) can be stopped and surveillance installed. The Guideline’s recommendation selected a trialed serological tool over skin snip for this specific activity on the basis of diagnostics characteristics and at a time when RDT had only just been available. In research recommendations, the Guidelines even advocated for validation of RDT as a possible replacement of the standard test, being ELISA, recognizing the potential of RDT even at the time it had not been widely trialed. The Guidelines were also very clear that tools for monitoring onchocerciasis intervention up to the point of demonstrating interruption of transmission, did not need to be restricted to Ov 16 ELISA.

 In the meantime, the WHO’s Onchocerciasis Technical Advisory Subgroup, since 2017, has been annually reviewing, among other things, the performance characteristics of two available Ov 16 ELISAs and RDT and provided clear recommendations on their uses based on technical capabilities. In addition, onchocerciasis diagnostic target product profile has been developed to guide development of the appropriate diagnostic tool required for use in onchocerciasis elimination process, as found in Biamonte *et al.* (2022 ^1^). This suggests that none of the available diagnostic tools is really very satisfactory. Finally, from informal conversations with African Programme Managers of onchocerciasis, who have not published their results in peer reviewed journals, all confirm that they use RDT for all situations in onchocerciasis intervention because of its simplicity in use, which suggest that this judgement might be based on a narrower view of events.

 It therefore appears evident that the statement is not accurate, judges the WHO unfairly and does not quite fit this place in the discussion. I think it should be deleted whilst the good attributes of the RDT remain standing.

Is the study design appropriate and does the work have academic merit?

Yes

Is the work clearly and accurately presented and does it cite the current literature?

Partly

If applicable, is the statistical analysis and its interpretation appropriate?

Yes

Are all the source data underlying the results available to ensure full reproducibility?

Yes

Are the conclusions drawn adequately supported by the results?

Yes

Are sufficient details of methods and analysis provided to allow replication by others?

Yes

Reviewer Expertise:

Onchocerciasis epidemiology

I confirm that I have read this submission and believe that I have an appropriate level of expertise to confirm that it is of an acceptable scientific standard.

## References

[1] : Onchocerciasis: Target product profiles of in vitro diagnostics to support onchocerciasis elimination mapping and mass drug administration stopping decisions. *PLoS Negl Trop Dis* .2022;16(8) : 10.1371/journal.pntd.0010682 e0010682 10.1371/journal.pntd.0010682 35921329 PMC9377578

# Reviewer report: Reviewer response for version 2

**DOI:** 10.21956/openreseurope.18760.r39102

**Published:** 2024-04-19

**Authors:** Norbert W Brattig

**Affiliations:** 1 Department Infectious Disease Epidemiology, Bernhard Nocht Institute for Tropical Medicine, Hamburg, Germany

I thank the authors for clarifying and correcting the manuscript according to the comments and critique of the reviewers. There are still some points in the revision which should be addressed - please see the attached file linked here for more information.

Is the study design appropriate and does the work have academic merit?

Yes

Is the work clearly and accurately presented and does it cite the current literature?

Yes

If applicable, is the statistical analysis and its interpretation appropriate?

I cannot comment. A qualified statistician is required.

Are all the source data underlying the results available to ensure full reproducibility?

Partly

Are the conclusions drawn adequately supported by the results?

Yes

Are sufficient details of methods and analysis provided to allow replication by others?

Yes

Reviewer Expertise:

Parasitology, microbiology, immunology, diagnostics, pathogen-host-interaction

I confirm that I have read this submission and believe that I have an appropriate level of expertise to confirm that it is of an acceptable scientific standard, however I have significant reservations, as outlined above.

# Reviewer report: Reviewer response for version 2

**DOI:** 10.21956/openreseurope.18760.r39103

**Published:** 2024-04-13

**Authors:** Kiswendsida Thierry Guiguemde

**Affiliations:** 1 Joseph Ki-Zerbo University, Ouagadougou, Burkina Faso

The authors worked on the importance of using rapid tests for the monitoring and control of onchocerciasis.

 The revised version of the manuscript has taken into account our comments and suggestions. This allows a good understanding of the survey procedures and the analysis of the data obtained.

 We have no further comments

Is the study design appropriate and does the work have academic merit?

Yes

Is the work clearly and accurately presented and does it cite the current literature?

Yes

If applicable, is the statistical analysis and its interpretation appropriate?

Yes

Are all the source data underlying the results available to ensure full reproducibility?

Yes

Are the conclusions drawn adequately supported by the results?

Yes

Are sufficient details of methods and analysis provided to allow replication by others?

Yes

Reviewer Expertise:

Parasitology

I confirm that I have read this submission and believe that I have an appropriate level of expertise to confirm that it is of an acceptable scientific standard.

# Reviewer report: Reviewer response for version 1

**DOI:** 10.21956/openreseurope.17376.r36629

**Published:** 2024-01-19

**Authors:** Yankum Dadzie

**Affiliations:** 1 Emory University, Atlanta, Georgia, USA

This is an interesting paper reporting on the effect of using a new onchocerciasis diagnostic tool to follow up an onchocerciasis intervention in an uncontrolled area, comparable to an onchocerciasis naïve area with a high endemicity. Every observation is thus intriguing whilst the interpretation of what is being observed needs to be done cautiously, particularly as regards the application of the new diagnostic tool.

 This study is well set out and appears well conducted. However, the paper must address two areas/points which constitute a setback for the paper’s strength. The first point is the use of some words or phrases in the paper which are not scientifically appropriate and give the wrong information on the situation in question. The second is about some points in the discussion of the results which portray an opinion which do not seem to be backed by evidence.

 The following concern comments on the first point with the use of inappropriate words or phrases:

 Introduction: Line 7-10, replace ‘Prevention’ with ‘Elimination’ and continue with the sentence and in the 10 ^th^ line substitute ‘control’ for ‘eradicate’.

 The last sentence of the introduction is confusing as what it states is not was what was done. I suggest the following formulation: “This paper presented the effect of strengthening the onchocerciasis elimination interventions as demonstrated by seroprevalence in children aged 3-9 years using the Ov16 SD BIOLINE rapid diagnostic test (RDT)”.

 Results:

 Under the subtitle ‘Ov16 seroprevalence in Maridi over time’, the following sentence in lines 3-5, ‘The seroprevalence among the three-year-olds, born after implementation of more robust onchocerciasis elimination measures like vector control and bi-annual CDTi, non-significantly decreased from 12.5% in 2019 to 8.8% in 2023 (p=0.68)’, needs to be reformulated. The three-year-olds born after implementation of more robust onchocerciasis elimination measures could be found only in 2023 and could therefore not have contributed to the RDT seroprevalence in 2019 to enable showing a reduced level in 2023. As the measurements are cross-sectional values the sentence should read correctly thus: The seroprevalence among three-year-olds, born after implementation of more robust onchocerciasis elimination measures like vector control and bi-annual CDTi measured in 2023 8% and was thus lower than the level of 12.5% measured among three-year-olds in 2019 but the difference was not significant, (p=0.68).

 My main concern is with the “discussion” and it is specifically about the interpretation of the results from the study. The first paragraph of the discussion points out the increase, albeit insignificant, of the seroprevalence in the 4–9-year-olds following enhanced onchocerciasis measures and interprets the ‘increased seroprevalence as 'an indication of intensification of transmission’. Such an interpretation, “increase in transmission” cannot be correct as the enhanced intervention measures clearly must have had increased effect entomologically as well as epidemiologically, through twice a year CDTi even if the coverage was not optimal yet, on the transmission. The entomological impact of “slash and clear” has been reported in the ref. no 5. The publication, “A force-of-infection model for onchocerciasis and its applications in the epidemiological evaluation of the Onchocerciasis Control Programme in the Volta River basin area by Remme, J *et al.* in the Bulletin of the World Health Organization, 64: 667-681 (1986) on which the onchocerciasis mathematical model is based, shows that at the onset of intervention of vector control, which interrupts transmission almost immediately, the mf prevalence of age groups in years 0-4, 5-9, continues to rise during the first five years after which it starts falling. It is only in the adults that there is a fall of mf prevalence which is seen in the first three years. A similar effect is found with CDTi where it is even more accentuated the higher the pre-intervention endemicity level of the community. It may be recalled that impact assessment of CDTi in APOC countries did not occur until after five years of CDTI when impact would be expected. The notion was based on this observation. Recently, Lont *et al. *have shown that there are basic similarities in the effect of application of mf prevalence and seroprevalence in their publication (Lont *et al.*, 2017 ^1^). The increase in the seroprevalence recorded in the age group of 3-9 years old children is normal and it is to be expected in that age group particularly when dealing with communities with very high pre-intervention endemicity. It may be noted that some of the young might harbour prepatent infection before the start of the intervention and which may break out in the course of the intervention as Lont *et al.* discuss in their paper.

 As regards to the above considerations, certain statements in the discussion will have to be reviewed and I would like to point them out:

 1 ^st^ paragraph, line 3-5 “These results suggest that the elimination measures did not significantly impact the seroprevalence of the study population”. The sentence may be deleted as what has been found is to be expected and importantly does not suggest an absence of impact. The continuation is correct as it takes a few years before interruption of transmission can occur showing also the absence of new infections or new seroconversions as applicable to CDTi.

 Line 10, “may indicate an intensification of *O. volvulus transmission”* may be deleted for the same reason as above.

 Paragraph 2 is well discussed. However, I would like to point out a striking mitigating factor which has either been downplayed or overseen. It is true that no CDTi was carried out in 2020 because of Covid-19 with the negative implications for which Hamley’s good work has been quoted to highlight the effect on this study. However, a good look at the first-year results following the first slash and clear activity as read from the paper by Raimon *et al.* suggests that Maridi focus possibly did not do as badly as it has been described in this paper. A proper analysis of the events could very well indicate that there could have been more reduction in transmission in the year 2020 effected by the slash and clear than what could have been achieved by annual CDTI alone before the slash and clear was introduced.

 In the penultimate line of the paragraph, kindly substitute ‘eliminate blackflies’ for ‘eradicate blackflies’.

 Paragraph 5:

 I wonder whether after the foregoing discussions it is still worth retaining this statement, particularly the second half of the statement. “A sufficiently large sample size of 3-year-old children needs to be Ov16 RDT tested before and after interventions to evaluate the short-term effect of strengthening an onchocerciasis elimination programme”.

Is the study design appropriate and does the work have academic merit?

Yes

Is the work clearly and accurately presented and does it cite the current literature?

Partly

If applicable, is the statistical analysis and its interpretation appropriate?

Yes

Are all the source data underlying the results available to ensure full reproducibility?

Yes

Are the conclusions drawn adequately supported by the results?

Yes

Are sufficient details of methods and analysis provided to allow replication by others?

Yes

Reviewer Expertise:

Onchocerciasis epidemiology

I confirm that I have read this submission and believe that I have an appropriate level of expertise to confirm that it is of an acceptable scientific standard, however I have significant reservations, as outlined above.

## References

[1] : Modelling Anti-Ov16 IgG4 Antibody Prevalence as an Indicator for Evaluation and Decision Making in Onchocerciasis Elimination Programmes. *PLoS Negl Trop Dis* .2017;11(1) : 10.1371/journal.pntd.0005314 e0005314 10.1371/journal.pntd.0005314 28114304 PMC5289624

# Response

**Published:** 2024-02-19

**Authors:** Amber Hadermann

**Affiliations:** Global Health institute, University of Antwerp, Antwerp, Flanders, Belgium

Response to the reviewer: Rapid diagnostic testing for onchocerciasis in Maridi (South Sudan) before and after improving elimination strategies: a repeated cross-sectional survey Reviewer

 1 : **Norbert Brattig **The study represents a local investigation on the success of onchocerciasis control endeavour in South Sudan. The authors analysed the antibody response in children to the diagnostic Onchocerca protein Ov16 in 2023 compared to 2019. The reported data indicate that despite the annual treatment with ivermectin (CDTI) and the vector control strategy “Slash and Clear” the Ov16 response did not decrease but showed insignificant increase in 4-9-year-old children (about 27% to 34%) but an insignificant decrease in 3-year-old children (about 12% to 9%). The cross-sectional survey indicates the ongoing Onchocerca transmission demanding strengthening of the onchocerciasis elimination programme.

 The work is clearly and accurately presented. The authors cited current references except the indications noted below. The study design is appropriate, the authors exhibit sufficient details of the analysis – except the constraint below – including the statistics and applied methods which in part should be better introduced. The article merits indexing for scientists involved in onchocerciasis control. The source data are available. The conclusions drawn adequately are supported by the results.

 However, the title appears not to represent the documented data as their conclusion and should be improved. Thus, not the applied diagnostic test pertains the core statement of the article, furthermore the notification “ *improving elimination strategies*” rather indicates a success in onchocerciasis control. The title more preferably should read: “ *Continuance of onchocerciasis transmission to children in a highly endemic area in South Sudan after ivermectin treatment and vector control* *” *or “ *Continuance of onchocerciasis transmission to children in the highly endemic Maridi county (South Sudan) after ivermectin treatment and vector control strategy requiring strengthening of onchocerciasis elimination programmes”*

***Response:* *We prefer to keep our title. Indeed, the aim of this paper was to report on the use of the OV16 rapid test to evaluate onchocerciasis elimination programs. In Maridi, because of the high prevalence of epilepsy and the high Ov16 seroprevalence among children, the onchocerciasis elimination program was already strengthened by switching to six monthly distribution of ivermectin and the implementation of the “Slash& Clear” programme. We now clarify this in the new introduction of the paper. This programme most likely decreased (but not yet interrupted) onchocerciasis transmission because we observed a decrease in the incidence of onchocerciasis-associated epilepsy, including nodding syndrome. These findings were recently published by Jada et al.* *Jada SR, Amaral LJ, Lakwo T, Carter JY, Rovarini J, Bol YY, Logora MY, Hadermann A, Hopkins A, Fodjo JNS, Colebunders R.* *Effect of onchocerciasis elimination measures on the incidence of epilepsy in Maridi, South Sudan: a 3-year longitudinal, prospective, population-based study.* *Lancet Glob Health. 2023 Aug;11(8):e1260-e1268.* *We now also mention this in the discussion of the paper and include this additional reference.*** ***We agree that the programme needs further strengthening as 8.8% of the 3-year-old children were still Ov16 positive. Our work also suggests Ov16 rapid testi**ng in 3-year olds as a potential tool to monitor changes in transmission over time.*    

**Objections:**

1. The authors should not state “ *Maridi Dam, the only blackfly breeding site in the area*” since this statement cannot be accurate, there surely are other breeding sites.

***Response:* *The rivers in the Maridi central area were extensively investigated for the presence of breeding sites by Lakwo et al. (2020), and the Maridi dam was found to be the single black fly breeding site in the area. The reference of the paper by Lakwo et al. is included in the reference list.  We agree this is a unique situation for an onchocerciasis endemic area. Our population-based studies showed that the Maridi Dam explains the reason why an “epilepsy epidemic” started in Maridi. Nevertheless, we now state “Maridi Dam, the major blackfly breeding site in the area”.*    **

1. The authors stated that the “a *nnual CDTi was introduced in South Sudan in the early 2000s”, *however, they in addition should refer to the detection and occurrence of onchocerciasis in Sudan *. *Thus, onchocerciasis had been reported in 1947 by Kirk, in 1985 by Williams et al..

 
***Response* *: We added the sentence:* *« Onchocerciasis was first reported in Sudan in 1947 by Kirk et al.* *»* *We also included the reference:* *Kirk R, Morgan HV, Haseeb MA, Satti MH. Onchocerciasis in the Sudan Republic. Ann Trop Med Parasitol. 1959;53(1):97-102.*  **

1. The authors stated the application of Ov16 rapid diagnostic tests by citing an earlier study of their research group, however, they should explain to the readers the significance and specificity of this *O. volvulus *antigen. Thus, *O. volvulus*-specific antibodies demonstrated in 1987 by Sisley et al., Ov16 antibodies by Higazi et al. in 2013 and 2016; Lobos et al. documented first the high specificity of Ov16 in 1991, later Richards et al. documented 2018 the specificity of the operational performance of *O. volvulus *Ov16 ELISA serological assay, followed by Weil et al. in 2000.

 
***Response* *: We have now added more information about the Ov16 test and included in the introduction:* *"Currently, the World Health Organisation (WHO) recommends using serodiagnostic tools based on the detection of antibodies specific to a 16KDa species-specific antigen of O. volvulus (Ov16), utilising either a rapid diagnostic test or an Enzyme-Linked Immunosorbent Assay (ELISA) platform. The antigen Ov16 serves as an early marker of onchocerciasis infection, so seroconversion to Ov16 can be used to detect pre-patent infection, which is not possible with skin snips. Since its discovery in 1991, the use of Ov16 has emerged as the gold standard for onchocerciasis serology, with sensitivities in the 80–89% range and specificity values in the 97–99% range depending on the assay used and the sample collection assessed.* *We also added the following references:* *World Health Organisation. Onchocerciasis: diagnostic target product profile to support preventive chemotherapy* *, 2021* *https://www.who.int/publications/i/item/9789240024496* *Lobos E, Altmann M, Mengod G, Weiss N, Rudin W, Karam M. Mol Biochem Parasitol.* *Identification of an Onchocerca volvulus cDNA encoding a low-molecular-weight antigen uniquely recognized by onchocerciasis patient sera.* *1990 Feb;39(1):135-45.* *Cama VA, McDonald C, Arcury-Quandt A, Eberhard M, Jenks MH, Smith J, et al. Evaluation of an OV-16 IgG4 Enzyme-Linked Immunosorbent Assay in Humans and Its Application to Determine the Dynamics of Antibody Responses in a Non-Human Primate Model of Onchocerca volvulus Infection. Am J Trop Med Hyg. 2018;99(4):1041-8.* *Golden A, Steel C, Yokobe L, Jackson E, Barney R, Kubofcik J, et al. Extended result reading window in lateral flow tests detecting exposure to Onchocerca volvulus: a new technology to improve epidemiological surveillance tools. PLoS One. 2013;8(7):e69231.*  **

1. The note of the recommendation of the Ov16 diagnostic test by the WHO in the text should include the respective reference (WHO, 2016 ^1^ ) **.**

 
*Response:* *reference added.*  

1. The author correctly accentuated the limitation of the presented study by (i) the small sample size (n=248), (ii) the short follow-up duration, and (iii) the impact of the interrupted ivermectin treatment by Covid-19 pandemic. Thus, this study needs follow-up survey.

 
***Response:* *Yes, we are planning a follow-up survey.***

**Reviewer 2: Kiswendsida Thierry Guiguemde **The authors worked on the importance of using rapid tests for the monitoring and control of onchocerciasis. Which is very interesting. However, a few comments could help improve the manuscript.

 Methods:

**Study procedures:**

 1. There were community mobilizers to spread the information. It would be good to specify who injected the children for the test and whether they were trained beforehand.  

***Response:*** ***We now included in the study procedure “Five local health care workers were trained to perform the Ov16 SD BIOLINE RDT and administer a questionnaire to the parents of the children.”***

**Results:**

**Ov16 seroprevalence in Maridi 2023**

 1. “During the 2023 *O. volvulus* seroprevalence study in Maridi, 248 children aged three to nine years were recruited. The median age was six years (IQR: 4-8), and 114 (46.0%) were males. Ninety-three (37.5%) of the participating children had so To make reading easier, it is good to harmonize the way you write the numbers. Either write in letters or write in numbers as for the 248 children. It must be me form of dermatitis, as evidenced by itching and/or visible skin lesions. Furthermore, four (1.6%) children were identified as having epilepsy2. “applied throughout the entire manuscript. Put the percentage after the number and the specimen. Example: Ninety-three of the participating children (37.5%) instead of "Ninety-three (37.5%) of the participating children". four children (1.6%) were identified instead of four (1.6%) children were identified.  

***Response: We have written the text to fit the following standards: “Spell out numbers one through nine, except in the case of units of measure or time. For these, and for values of 10 and higher, use Arabic numerals. Always spell out numbers at the beginning of a sentence if the sentence cannot be rearranged to avoid starting with a number.” -* *Springer* *. Therefore, we have chosen to keep our way of writing numbers the same throughout this document.* *However, we have adapted the placement of percentages as suggested.***

 2. The overall Ov16 RDT seroprevalence was 76/248 (30.7%). The overall Ov16 RDT seroprevalence was 76/248 (30.7%).

 The overall Ov16 RDT seroprevalence is in percentage, putting 30.7% (76/248). Correct everywhere in the text the way of writing.  

***Response:* *We have adapted the text to fit the suggested standards.***

**Discussion:**

 1.  A significantly higher proportion of children with dermatitis tested Ov16 RDT positive (43.5%) compared to their peers without dermatitis (22.6%, p<0.001), suggesting the dermatitis was likely caused by an active *O. volvulus* infection in a large proportion of children.

 Other causes of dermatitis in children in the study area must be clarified and discussed.

***Response:* *We now include in the discussion: “In resource-constrained settings, when African children present with dermatitis and itching, scabies should be considered as a possible diagnosis. However, the absence of characteristic scabies lesions was noted in the children included in the study. Additionally, since scabies is treatable with ivermectin, its prevalence typically decreases in regions where mass ivermectin distribution occurs.”***

 2. More elements of discussion should be provided. Studies have shown limitations in the detection of antibodies against the OV-16 antigen. This is after discrepancies in the results with skin snip method according to WHO standards for the detection of microfilariae in the blood. It is important to specify this and give possible reasons. Carrying out the skin snip method in children over 5 with dermatitis could provide more precision on the cause of dermatitis.  

***Response:* *Indeed, the addition of skin snips would have added more information on active disease in the children. However, skin snip testing is increasingly not very well accepted by local populations because it is an invasive procedure. The use of rapid diagnostic tests is not to diagnose an O. volvulus infection on an individual level but to identify whether transmission is ongoing or not. Furthermore, in the age of ivermectin, the use of skin snip microscopy is becoming redundant. The number of microfilariae in the skin after ivermectin use is low and often below the detection limit. Finally, the fact that Ov16 antibodies can be detected even before the production of microfilariae make it a much better proxy for recent onchocerciasis transmission as it can identify pre-patent infections in recently infected children.***

**Reviewer 3 : Yankum Dadzie **This is an interesting paper reporting on the effect of using a new onchocerciasis diagnostic tool to follow up an onchocerciasis intervention in an uncontrolled area, comparable to an onchocerciasis naïve area with a high endemicity. Every observation is thus intriguing whilst the interpretation of what is being observed needs to be done cautiously, particularly as regards the application of the new diagnostic tool.

 This study is well set out and appears well conducted. However, the paper must address two areas/points which constitute a setback for the paper’s strength. The first point is the use of some words or phrases in the paper which are not scientifically appropriate and give the wrong information on the situation in question. The second is about some points in the discussion of the results which portray an opinion which do not seem to be backed by evidence.

1. The following concern comments on the first point with the use of inappropriate words or phrases:
  Introduction: Line 7-10, replace ‘Prevention’ with ‘Elimination’ and continue with the sentence and in the 10 ^th^ line substitute ‘control’ for ‘eradicate’.
  The last sentence of the introduction is confusing as what it states is not was what was done. I suggest the following formulation: “This paper presented the effect of strengthening the onchocerciasis elimination interventions as demonstrated by seroprevalence in children aged 3-9 years using the Ov16 SD BIOLINE rapid diagnostic test (RDT)”.

 
***Response: Suggested alterations done.*  **

1. Results:
  Under the subtitle ‘Ov16 seroprevalence in Maridi over time’, the following sentence in lines 3-5, ‘The seroprevalence among the three-year-olds, born after implementation of more robust onchocerciasis elimination measures like vector control and bi-annual CDTi, non-significantly decreased from 12.5% in 2019 to 8.8% in 2023 (p=0.68)’, needs to be reformulated. The three-year-olds born after implementation of more robust onchocerciasis elimination measures could be found only in 2023 and could therefore not have contributed to the RDT seroprevalence in 2019 to enable showing a reduced level in 2023. As the measurements are cross-sectional values the sentence should read correctly thus: The seroprevalence among three-year-olds, born after implementation of more robust onchocerciasis elimination measures like vector control and bi-annual CDTi measured in 2023 8% and was thus lower than the level of 12.5% measured among three-year-olds in 2019 but the difference was not significant, (p=0.68).

***Response: Text altered. We now state”* *The seroprevalence among three-year-olds, born after implementation of more robust onchocerciasis elimination measures like vector control and bi-annual CDTi, was measured at 8% in 2023 and was thus lower than the level of 12.5% measured among three-year-olds in 2019. However, the difference was not significant (p=0.68).”***

1. My main concern is with the “discussion” and it is specifically about the interpretation of the results from the study. The first paragraph of the discussion points out the increase, albeit insignificant, of the seroprevalence in the 4–9-year-olds following enhanced onchocerciasis measures and interprets the ‘increased seroprevalence as 'an indication of intensification of transmission’. Such an interpretation, “increase in transmission” cannot be correct as the enhanced intervention measures clearly must have had increased effect entomologically as well as epidemiologically, through twice a year CDTi even if the coverage was not optimal yet, on the transmission. The entomological impact of “slash and clear” has been reported in the ref. no 5. The publication, “A force-of-infection model for onchocerciasis and its applications in the epidemiological evaluation of the Onchocerciasis Control Programme in the Volta River basin area by Remme, J *et al.* in the Bulletin of the World Health Organization, 64: 667-681 (1986) on which the onchocerciasis mathematical model is based, shows that at the onset of intervention of vector control, which interrupts transmission almost immediately, the mf prevalence of age groups in years 0-4, 5-9, continues to rise during the first five years after which it starts falling. It is only in the adults that there is a fall of mf prevalence which is seen in the first three years. A similar effect is found with CDTi where it is even more accentuated the higher the pre-intervention endemicity level of the community. It may be recalled that impact assessment of CDTi in APOC countries did not occur until after five years of CDTI when impact would be expected. The notion was based on this observation. Recently, Lont *et al. *have shown that there are basic similarities in the effect of application of mf prevalence and seroprevalence in their publication (Lont *et al.*, 2017 ^1^ ). The increase in the seroprevalence recorded in the age group of 3-9 years old children is normal and it is to be expected in that age group particularly when dealing with communities with very high pre-intervention endemicity. It may be noted that some of the young might harbour prepatent infection before the start of the intervention and which may break out in the course of the intervention as Lont *et al.* discuss in their paper.

  As regards to the above considerations, certain statements in the discussion will have to be reviewed and I would like to point them out:

 1 ^st^ paragraph, line 3-5 “These results suggest that the elimination measures did not significantly impact the seroprevalence of the study population”. The sentence may be deleted as what has been found is to be expected and importantly does not suggest an absence of impact. The continuation is correct as it takes a few years before interruption of transmission can occur showing also the absence of new infections or new seroconversions as applicable to CDTi. *Response: We thank you for these important comments. We eliminated the sentence  “These results suggest that the elimination measures did not significantly impact the seroprevalence of the study population”.* *We now included in the discussion “An increase in the seroprevalence in the age group of 3-9 years old children was to be expected, particularly because of the very high pre-intervention endemicity. Indeed some of the children might harbour prepatent infection before the start of the intervention which may break out in the course of the intervention. A force-of-infection model for onchocerciasis has shown that even during interruption of O. volvulus transmission by vector control, the microfilarial prevalence of the 0-4 and 5-9 year old age groups continues to rise during the first five years after which it starts falling.”* *And we added the following two references* *Lont YL, Coffeng LE, de Vlas SJ, Golden A, de Los Santos T, Domingo GJ, Stolk WA. PLoS Negl Trop Dis. 2017 Jan 23;11(1):e0005314. doi: 10.1371/journal.pntd.0005314. eCollection 2017* *Remme J, Ba O, Dadzie KY, Karam M. A force-of-infectionmodel for onchocerciasis and its applications in the epidemiological evaluation of the Onchocerciasis Control Programme in the Volta River basin area. Bull World Health Organ. 1986;64(5):667-81*  

1. Line 10, “may indicate an intensification of *O. volvulus transmission” *may be deleted for the same reason as above.

 
***Response: We deleted the the text mentioning the ‘ *intensification of *O. volvulus transmission” * **

1. Paragraph 2 is well discussed. However, I would like to point out a striking mitigating factor which has either been downplayed or overseen. It is true that no CDTi was carried out in 2020 because of Covid-19 with the negative implications for which Hamley’s good work has been quoted to highlight the effect on this study. However, a good look at the first-year results following the first slash and clear activity as read from the paper by Raimon *et al.* suggests that Maridi focus possibly did not do as badly as it has been described in this paper. A proper analysis of the events could very well indicate that there could have been more reduction in transmission in the year 2020 effected by the slash and clear than what could have been achieved by annual CDTI alone before the slash and clear was introduced.

 
***Response: We agree that the strengthening of the onchocerciasis elimination program decreased the O. volvulus transmission in the area. This was strongly suggested by the results of a longitudinal population-based study that showed that the incidence of onchocerciasis-associated epilepsy, including nodding syndrome, decreased in Maridi. This paper has now been published and is included among the references.* *Jada SR, Amaral LJ, Lakwo T, Carter JY, Rovarini J, Bol YY, Logora MY, Hadermann A, Hopkins A, Fodjo JNS, Colebunders R. Effect of onchocerciasis elimination measures on the incidence of epilepsy in Maridi, South Sudan: a 3-year longitudinal, prospective, population-based study. Lancet Glob Health. 2023 Aug;11(8):e1260-e1268. doi: 10.1016/S2214-109X(23)00248-*6.  **

1. In the penultimate line of the paragraph, kindly substitute ‘eliminate blackflies’ for ‘eradicate blackflies’.

 
*Response: Text altered.*    

1. Paragraph 5:
  I wonder whether after the foregoing discussions it is still worth retaining this statement, particularly the second half of the statement. “A sufficiently large sample size of 3-year-old children needs to be Ov16 RDT tested before and after interventions to evaluate the short-term effect of strengthening an onchocerciasis elimination programme”.

 
*Response: We now state “A sufficiently large sample size of 3-year-old children needs to be Ov16 RDT tested and a longer follow-up is needed to investigate the  effect of strengthening an onchocerciasis elimination programme.”*

# Reviewer report: Reviewer response for version 1

**DOI:** 10.21956/openreseurope.17376.r36626

**Published:** 2024-01-19

**Authors:** Kiswendsida Thierry Guiguemde

**Affiliations:** 1 Joseph Ki-Zerbo University, Ouagadougou, Burkina Faso

The authors worked on the importance of using rapid tests for the monitoring and control of onchocerciasis. Which is very interesting. However, a few comments could help improve the manuscript.

 Methods:

**Study procedures:**

 1. There were community mobilizers to spread the information. It would be good to specify who injected the children for the test and whether they were trained beforehand.

**Results:**

**Ov16 seroprevalence in Maridi 2023**

 1. “During the 2023 O. volvulus seroprevalence study in Maridi, 248 children aged three to nine years were recruited. The median age was six years (IQR: 4-8), and 114 (46.0%) were males. Ninety-three (37.5%) of the participating children had some form of dermatitis, as evidenced by itching and/or visible skin lesions. Furthermore, four (1.6%) children were identified as having epilepsy2. “

- To make reading easier, it is good to harmonize the way you write the numbers. Either write in letters or write in numbers as for the 248 children. It must be applied throughout the entire manuscript.
- Put the percentage after the number and the specimen. Example: Ninety-three of the participating children (37.5%) instead of "Ninety-three (37.5%) of the participating children". four children (1.6%) were identified instead of four (1.6%) children were identified.

2. The overall Ov16 RDT seroprevalence was 76/248 (30.7%). The overall Ov16 RDT seroprevalence was 76/248 (30.7%).

 The overall Ov16 RDT seroprevalence is in percentage, putting 30.7% (76/248). Correct everywhere in the text the way of writing.

**Discussion:**

 1.  A significantly higher proportion of children with dermatitis tested Ov16 RDT positive (43.5%) compared to their peers without dermatitis (22.6%, p<0.001), suggesting the dermatitis was likely caused by an active *O. volvulus* infection in a large proportion of children.

 Other causes of dermatitis in children in the study area must be clarified and discussed.

 2. More elements of discussion should be provided. Studies have shown limitations in the detection of antibodies against the OV-16 antigen. This is after discrepancies in the results with skin snip method according to WHO standards for the detection of microfilariae in the blood. It is important to specify this and give possible reasons. Carrying out the skin snip method in children over 5 with dermatitis could provide more precision on the cause of dermatitis.

Is the study design appropriate and does the work have academic merit?

Yes

Is the work clearly and accurately presented and does it cite the current literature?

Yes

If applicable, is the statistical analysis and its interpretation appropriate?

Yes

Are all the source data underlying the results available to ensure full reproducibility?

Yes

Are the conclusions drawn adequately supported by the results?

Yes

Are sufficient details of methods and analysis provided to allow replication by others?

Yes

Reviewer Expertise:

Parasitology

I confirm that I have read this submission and believe that I have an appropriate level of expertise to confirm that it is of an acceptable scientific standard, however I have significant reservations, as outlined above.

# Reviewer report: Reviewer response for version 1

**DOI:** 10.21956/openreseurope.17376.r36322

**Published:** 2024-01-19

**Authors:** Norbert W Brattig

**Affiliations:** 1 Department Infectious Disease Epidemiology, Bernhard Nocht Institute for Tropical Medicine, Hamburg, Germany

The study represents a local investigation on the success of onchocerciasis control endeavour in South Sudan. The authors analysed the antibody response in children to the diagnostic *Onchocerca *protein Ov16 in 2023 compared to 2019. The reported data indicate that despite the annual treatment with ivermectin (CDTI) and the vector control strategy “Slash and Clear” the Ov16 response did not decrease but showed insignificant increase in 4-9-year-old children (about 27% to 34%) but an insignificant decrease in 3-year-old children (about 12% to 9%). The cross-sectional survey indicates the ongoing *Onchocerca *transmission demanding strengthening of the onchocerciasis elimination programme.

 The work is clearly and accurately presented. The authors cited current references except the indications noted below. The study design is appropriate, the authors exhibit sufficient details of the analysis – except the constraint below – including the statistics and applied methods which in part should be better introduced. The article merits indexing for scientists involved in onchocerciasis control. The source data are available. The conclusions drawn adequately are supported by the results.

 However, the title appears not to represent the documented data as their conclusion and should be improved. Thus, not the applied diagnostic test pertains the core statement of the article, furthermore the notification “ *improving elimination strategies*” rather indicates a success in onchocerciasis control. The title more preferably should read: “ *Continuance of onchocerciasis transmission to children in a highly endemic area in South Sudan after ivermectin treatment and vector control” *or “ *Continuance of onchocerciasis transmission to children in the highly endemic Maridi county (South Sudan) after ivermectin treatment and vector control strategy requiring strengthening of onchocerciasis elimination programmes”*

 Objections:

- The authors should not state “ *Maridi Dam, the only blackfly breeding site in the area*” since this statement cannot be accurate, there surely are other breeding sites.
- The authors stated that the “a *nnual CDTi was introduced in South Sudan in the early 2000s”, *however, they in addition should refer to the detection and occurrence of onchocerciasis in Sudan *. *Thus, onchocerciasis had been reported in 1947 by Kirk, in 1985 by Williams et al..
- The authors stated the application of Ov16 rapid diagnostic tests by citing an earlier study of their research group, however, they should explain to the readers the significance and specificity of this *O. volvulus *antigen. Thus, *O. volvulus*-specific antibodies demonstrated in 1987 by Sisley et al., Ov16 antibodies by Higazi et al. in 2013 and 2016; Lobos et al. documented first the high specificity of Ov16 in 1991, later Richards et al. documented 2018 the specificity of the operational performance of *O. volvulus *Ov16 ELISA serological assay, followed by Weil et al. in 2000.
- The note of the recommendation of the Ov16 diagnostic test by the WHO in the text should include the respective reference (WHO, 2016 ^1^) **.**
- The author correctly accentuated the limitation of the presented study by (i) the small sample size (n=248), (ii) the short follow-up duration, and (iii) the impact of the interrupted ivermectin treatment by Covid-19 pandemic. Thus, this study needs follow-up survey.

In summary, this article should be indexed after minor revision:                                                          

 Decision: approved with minor reservation.

Is the study design appropriate and does the work have academic merit?

Yes

Is the work clearly and accurately presented and does it cite the current literature?

Yes

If applicable, is the statistical analysis and its interpretation appropriate?

I cannot comment. A qualified statistician is required.

Are all the source data underlying the results available to ensure full reproducibility?

Partly

Are the conclusions drawn adequately supported by the results?

Yes

Are sufficient details of methods and analysis provided to allow replication by others?

Yes

Reviewer Expertise:

Parasitology, microbiology, immunology, diagnostics, pathogen-host-interaction

I confirm that I have read this submission and believe that I have an appropriate level of expertise to confirm that it is of an acceptable scientific standard, however I have significant reservations, as outlined above.

## References

[1] Guidelines for Stopping Mass Drug Administration and Verifying Elimination of Human Onchocerciasis: Criteria and Procedures.2016; 26913317
